# Retraction: MicroRNA-138 acts as a tumor suppressor in non small cell lung cancer via targeting YAP1

**DOI:** 10.18632/oncotarget.28645

**Published:** 2024-08-26

**Authors:** Ling Xiao, Hui Zhou, Xiang-Ping Li, Juan Chen, Chao Fang, Chen-Xue Mao, Jia-Jia Cui, Wei Zhang, Hong-Hao Zhou, Ji-Ye Yin, Zhao-Qian Liu

**Affiliations:** ^1^Department of Clinical Pharmacology, Xiangya Hospital, Central South University, Hunan 410078, P.R. China; ^2^Institute of Clinical Pharmacology, Central South University, Hunan Key Laboratory of Pharmacogenetics, Changsha, Hunan 410078, P.R. China; ^3^Department of Histology and Embryology, School of Basic Medical Sciences, Central South University, Changsha, Hunan 410013, P.R. China; ^4^The Affiliated Cancer Hospital, XiangYa School of Medicine, Central South University, Changsha, Hunan 410014, P.R. China

**This article has been retracted:** There is internal duplication in Figures 2C and 4C. The same image was found to be duplicated in four other papers: Figures 4 and 7 from [1], Figure 3C, 3D [2], Figure 4C from [3]; and Figures 3B and 6B from [4].

Figure 4A duplicates Figure 5A from [5] and Figure 3G from [6]. The authors acknowledged the misuse of the images. The Scientific Integrity Committee of Xyangui Hospital investigated the paper and agreed with the retraction. As a result, the Editorial decision was made to retract this paper. All authors agreed with the decision.

Original article: Oncotarget. 2016; 7:40038–40046. 40038-40046. https://doi.org/10.18632/oncotarget.9480
